# Sequential ruptures of penetrating atherosclerotic ulcers of ascending aorta, aortic arch and descending thoracic aorta

**DOI:** 10.1186/s13019-020-01311-y

**Published:** 2020-10-06

**Authors:** Pankaj Kaul, Rodolfo Paniagua, Afroditi Petsa, Raj Singh

**Affiliations:** 1 Cardiac Surgeon Leeds General Infirmary, Leeds, LS1 3EX UK; 2 Department of Cardiac Surgery, Leeds General Infirmary, Leeds, LS1 3EX UK; 3 Department of Cardiac Anaesthesia, Leeds General Infirmary, Leeds, LS1 3EX UK

**Keywords:** Penetrating aortic ulcer, Rupture, Ascending aorta, Aortic arch, Descending thoracic aorta, Pseudoaneurysm

## Abstract

**Background:**

Penetrating ulcers of aorta, aortic dissections and intramural hematomas all come under acute aortic syndromes and have important similarities and differences.

**Case report:**

We report a 67 year old man with rupture of a large penetrating ulcer of the distal ascending aorta with hemopericardium and left hemothorax. He underwent interposition graft replacement of ascending aorta and hemi-arch with a 30 mm Gelweave Vascutek graft but represented 6 months later with development of a penetrating ulcer which ruptured into a huge 14 cm pseudoaneurysm. This was repaired with a 28 mm Vascutek Gelseal graft replacement of arch and interposition graft reconstruction of innominate and left common carotid arteries. 6 weeks later, however, he ruptured his proximal descending aorta and underwent TEVAR satisfactorily. Unfortunately, 2 days later, he developed a pathological fracture of left proximal tibia with metastasis from a primary renal cell carcinoma. He died 3 weeks later from respiratory failure.

We shall briefly outline the similarities and differences in presentation and management of penetrating aortic ulcers, aortic dissections and intramural haematomas. We shall discuss, in greater detail, penetrating ulcers of thoracic aorta, their natural history, location, complications and management.

**Conclusion:**

This case report is unique on account of initial successful surgical redressal following rupture of penetrating ulcer of distal ascending aorta into left pleural and pericardial cavities, normally associated with instant death. The haemodynamic effects of the rupture were staggered due to initial contained rupture into a smaller pseudoaneurysm, followed by a further rupture into a false aneurysmal sac followed eventually by generalised rupture into the pleural and pericardial cavities - a unique way of aortic rupture. Further development of another penetrating ulcer and a small pseudoaneurysm in the distal arch 6 months later which further ruptured into a larger 14 cm false aneurysmal sac, which again did not result in exsanguination, is again extraordinarily rare. Thereafter he underwent emergency thoracic endovascular aortic repair (TEVAR) for a further rupture of descending thoracic aorta. All three ruptures were managed successfully and would usually be associated with near-certain death, only for the patient to succumb eventually to the complications of metastatic renal cell carcinoma.

## Background

Four different pathological variants are usually summarised under the term “acute aortic syndrome” (AAS): aortic dissection (AD), intramural aortic hematoma (IMH), penetrating aortic ulcer (PAU) and contained aortic rupture (CAR).

Coady and Elefteriades summarised the important differences in the first three pathological variants [1]. Aortic dissections involve a flap which traverses the aortic lumen. IMH and PAU are non-flap entities and comprise 8% of acute aortic syndromes. IMH and PAU usually manifest in older patients in 7th, 8th and 9th decades of life. These patients are invariably hypertensive (94%). Unlike classic aortic dissections, PAU and IMH do not produce branch vessel compromise or occlusion and do not produce ischaemic manifestations in extremities or visceral organs. PAU thus is a focal lesion, and demonstrates a crater extending from the aortic lumen into the space surrounding the aortic lumen. It does not spread longitudinally. It is often associated with severe aortic atherosclerosis and calcification, whereas classic aortic dissection often involves aortas without significant calcification or arteriosclerosis. Both PAU and IMH tend to occur in larger aortas than in classic dissections and both are associated with a high incidence of concomitant AAA (42.1% in PAU). Both PAU and IMH are predominantly diseases of descending aorta (90 and 71% respectively) and behave much more malignantly than a typical type B dissection. The rarer PAU of ascending aorta and arch leads to dissection and rupture in 57% patients compared to only 14% in descending aorta. Type A PAU is thus primarily considered for surgical management and type B PAU, unless there are signs of instability, for medical management [1].

Rupture of PAU is a terminal event and there are anecdotal reports of survivors with contained rupture but none with rupture into both pleural and pericardial cavities.

## Case report

**Fig. 1:**
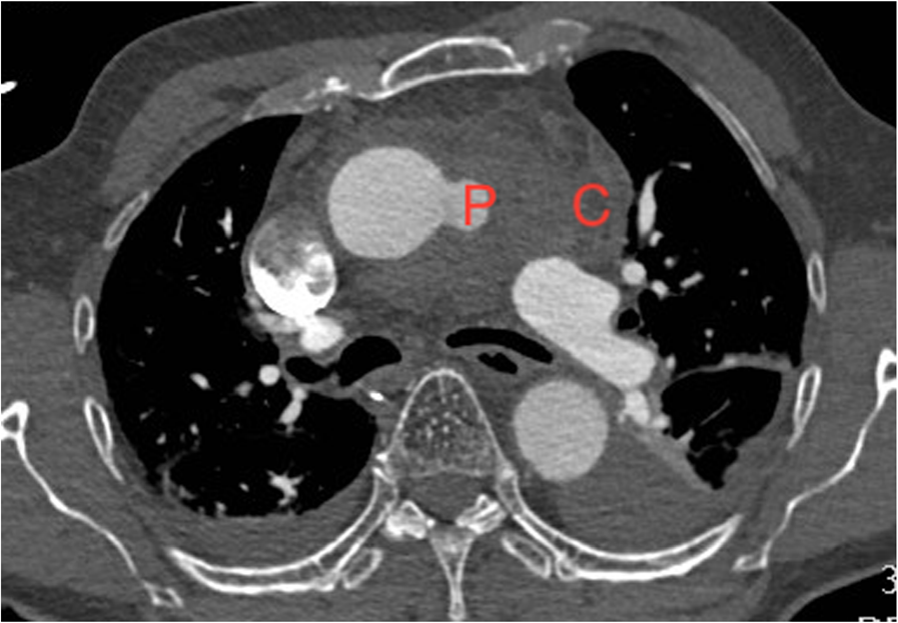
CT scan showing large pseudoaneurysm (P) in relation to the distal ascending aorta with rupture into adjoining soft tissue with considerable walled mediastinal clot (C) and left hemothorax

A 67 years old man presented in extremis with severe shortness of breath, in fast atrial fibrillation, with ST elevation in inferior leads and cardiogenic shock. Coronary angiogram demonstrated no significant coronary artery disease. Chest X-ray showed mediastinal widening and left haemothorax. CT scan revealed a large penetrating atherosclerotic ulcer at the junction of the distal ascending aorta and the undersurface of aortic arch, a pseudoaneurysm in relation to the distal ascending aorta, a large encapsulated collection of blood and clot surrounding the pseudoaneurysm and left hemothorax and hemopericardium (Fig. 1). Echocardiogram showed normal aortic valve, aortic root and proximal ascending aorta, mild MR with bileaflet prolapse, grossly abnormal-looking distal ascending aorta with a large pseudoaneurysm in relation to the distal ascending aorta and fair left ventricular contractility.

**Fig. 2:**
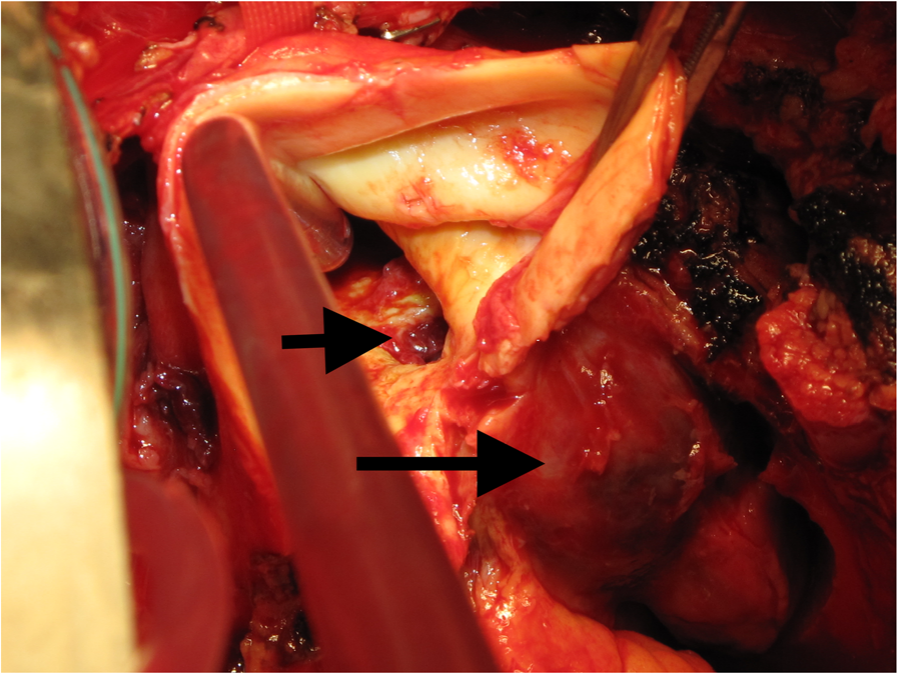
Intraoperative picture showing the large (small arrow) and smaller multiple penetrating ulcers of the distal ascending aorta, the pseudoaneurysm (large arrow) and the fibrous cavity into which the pseudoaneurysm had burst

At operation, distal ascending aorta was diffusely atheromatous. There was a large penetrating ulcer measuring 2 cm × 1.5 cm at the junction of ascending aorta and undersurface of arch which had ruptured into a pseudoaneurysm which in turn ruptured into a large aneurysmal sac which had further ruptured into both left pleural and pericardial cavities with about a litre of fresh blood and clot (Fig. 2). There were three smaller ulcers in the undersurface of the proximal arch. Cardiopulmonary bypass was instituted using a 22 Eiopa cannula advanced into the aortic arch from the innominate artery and right atrial venous drainage. We described this technique of arterial cannulation in acute aortic dissections in 2012. At 17 degree C, aorta was cross clamped, horizontal aortotomy made and heart arrested with 1 L antegrade coronary ostial cardioplegia. The ascending aorta was transected just above the sinotubular junction (STJ) and a 30 mm Gelweave Vascutek graft sutured in place using 5 “O” Prolene. The innominate cannula was now withdrawn from the arch and redirected into innominate artery to provide uni-hemispherical antegrade selective cerebral perfusion (ASCP) at 1 to 1.5 L/min and the cranial and left subclavian arterial tapes were snugged down at 17 d C with bilateral good cerebral oximetry traces. Aortic cross clamp was removed and the whole of the remaining ascending aorta and the entire under surface of the arch, including the large penetrating ulcer and the smaller three distal ulcers, were excised and the distal end of the Vascutek graft anastomosed to the remaining healthy under-surface of the arch with 5 “O” Prolene. Innominate cannula was repositioned into the arch thereafter, full cardiopulmonary bypass re-established. After rewarming and deairing, cardiopulmonary bypass was discontinued uneventfully. Excised aorta was reported on histopathology to show generalised atherosclerosis. Patient was discharged home after 10 days, having made an uncomplicated recovery.

**Fig. 3:**
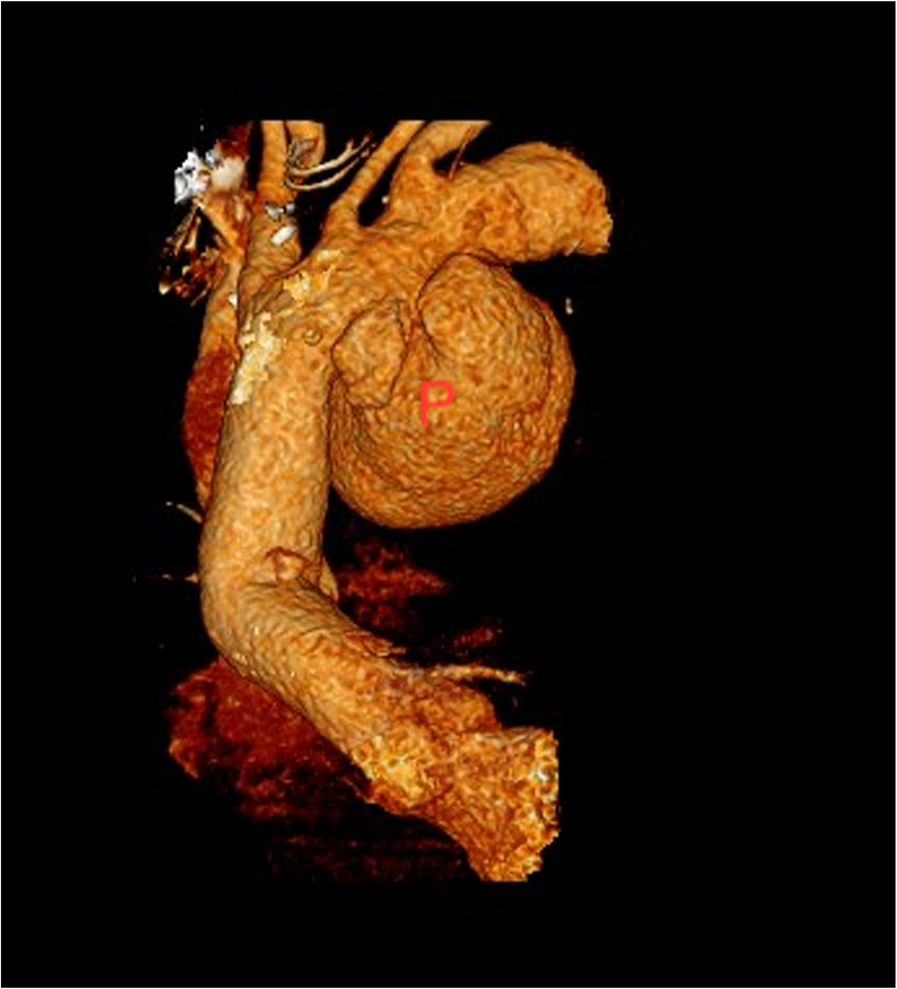
CT scan showing 14 cm pseudoaneurysm arising from the arch of aorta (P)

He remained well for 6 months but represented with worsening shortness of breath over the previous two weeks, mild chest pain and hoarseness of voice. Computerised tomography (CT) scan showed a 14 cm pseudoaneurysm in relation to the aortic arch (Fig. 3).

**Fig. 4:**
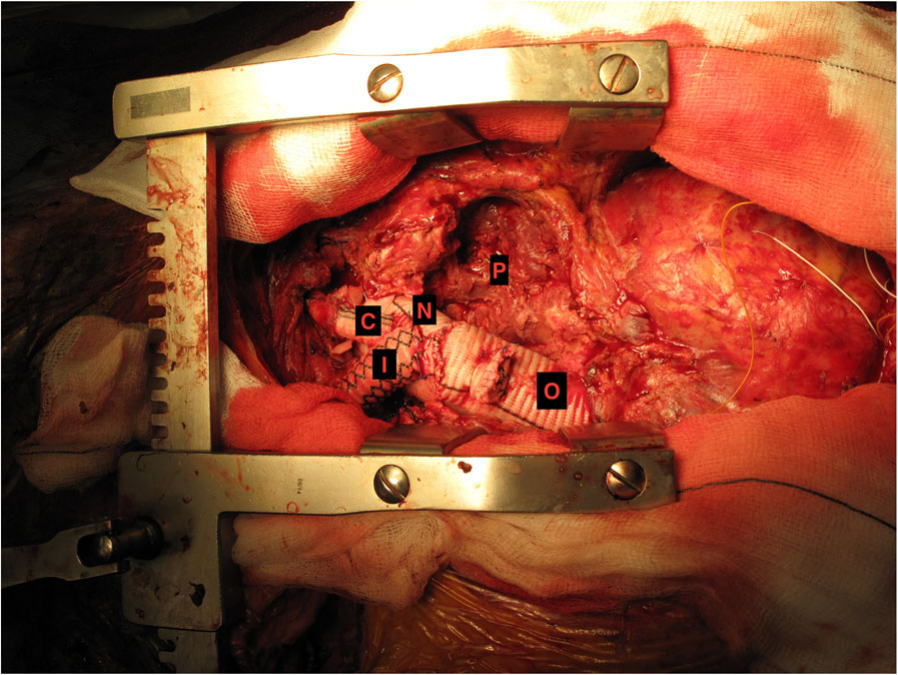
Intraoperative picture showing arch replacement using dacron graft for the arch (N) and 12 and 8 mm interposition PTFE grafts for the innominate (I) and left common carotid (C) arteries. The old graft replacing the ascending aorta (O)and the pseudoaneurysmal cavity laid open (P) is seen as well

He underwent a redo sternotomy. Cardiopulmonary bypass was established with composite right axillary and right femoral arterial return and right femoral venous drainage and secondary median sternotomy made. Heart and great vessels were dissected out. There was a 14 cm pseudoaneurysm filled with clot and debris, compressing the main pulmonary artery, in relation to the distal arch. The left brachiocephalic vein was divided for adequate exposure of the pseudoaneurysm and the arch vessels. Patient was cooled to 17 d C and uni-hemispherical ASCP at 1.5 L established through the right axillary artery. The distal arch, well beyond the previous anastomosis, had ruptured along half of its circumference into the pseudoaneurysm. The aortic arch was excised and the origins of innominate artery (IA) and left common carotid artery (LCCA) were divided. The left subclavian artery (LSA) arising somewhat distally from the proximal descending aorta was left attached to the proximal descending aorta. A 28 mm Vascutek dacron graft was anastomosed distally to the descending aorta with bovine pericardial buttresses and the proximal end anastomosed to the distal end of the old 30 mm Vascutek graft. The IA and the LCCA were anastomosed to the 28 mm Vascutek graft with 12 and 8 mm Vascutek interposition grafts (Fig. 4) and whole-body cardiopulmonary bypass recommenced. The false aneurysm cavity was extensively debrided and laid open. After rewarming, bypass was discontinued easily.

He required gradual respiratory weaning, on account of pneumonia and general frailty, necessitating a percutaneous tracheostomy and antibiotics.

**Fig. 5:**
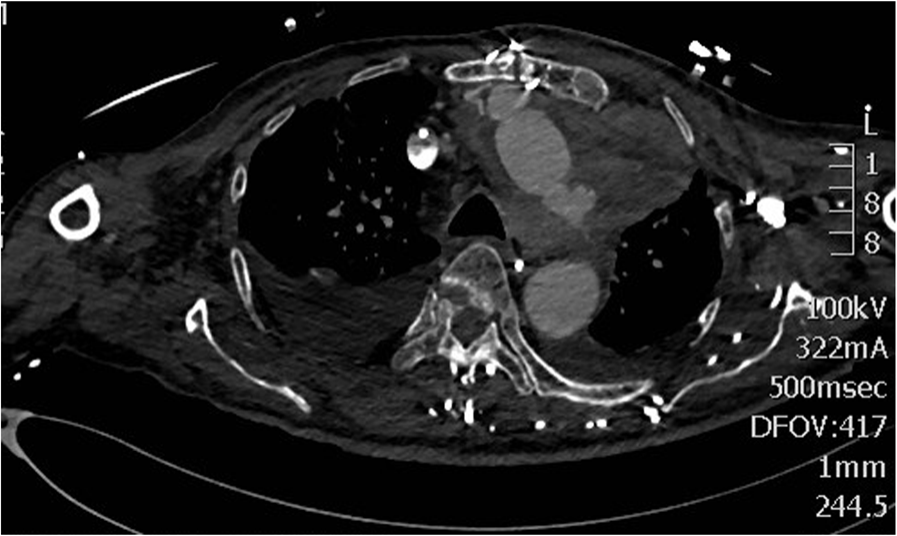
CT scan showing large leak with huge hematoma around proximal descending aorta

**Fig. 6:**
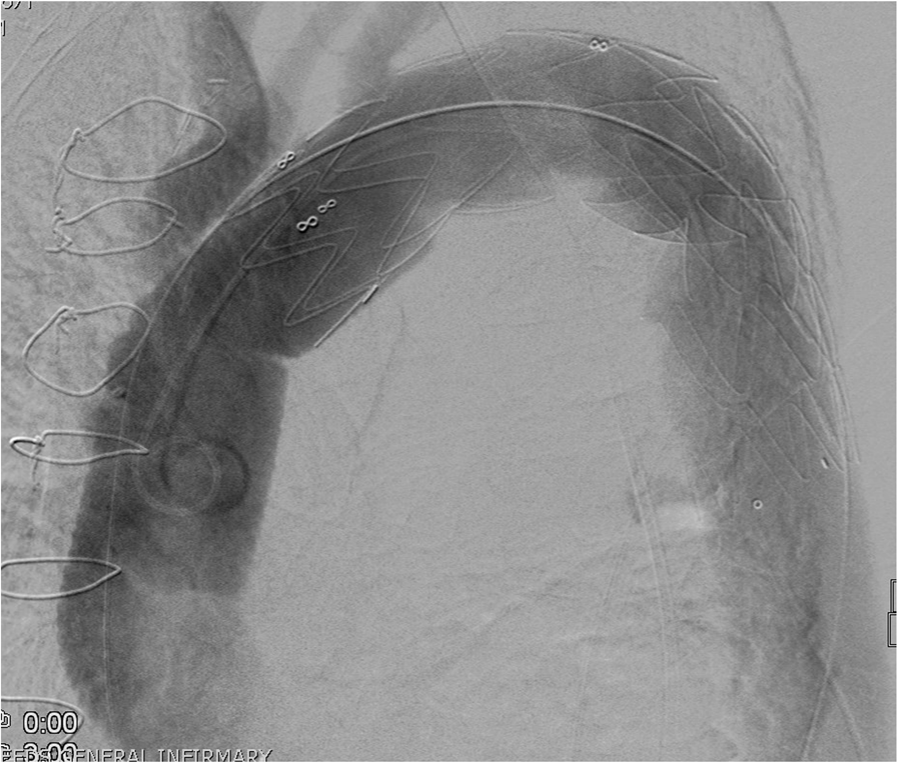
CT scan showing TEVAR of descending thoracic aorta

This, however, was interrupted suddenly, 6 weeks after his second operation, by sudden hypotension and respiratory distress when he was found to have, on an emergency CT scan, a rupture and contrast leak of the descending thoracic aorta well-beyond the distal anastomotic site with a large hematoma (Fig. 5). He underwent TEVAR with an excellent result (Fig. 6).

2 days later he developed swelling of left knee which was shown to be a pathological fracture of left proximal tibia. A staging scan showed an osteolytic lesion and pathological fracture of the medial cortex of left tibia, a metastatic lesion in the right neck of femur and a 3.4 cm right renal lesion, possibly a renal cell carcinoma. In view of his general frailty, cachexia, poor functional status and respiratory reserve on the background of metastatic renal cancer and pathological limb fractures, he was referred to the palliative care team. He continued to deteriorate globally, 65 days following his total arch replacement and it was clear he was coming to the end of his life. He passed away peacefully on the same day.

## Discussion and conclusion

Acute aortic syndrome, in contradistinction to aneurysmal aortic disease, presents with chest pain at its initial presentation. Three distinct pathological entities are lumped together in this non-specific term and, for this reason alone, it should perhaps not be used.

Aortic dissection (AD) involves a flap which traverses the aortic lumen and starts in an intimal tear [1].

Intramural hematoma of the thoracic aorta (IMH) is a diagnosis of exclusion and represents spontaneous, localised haemorrhage into the wall of thoracic aorta, usually in hypertensive and atherosclerotic patients, in the absence of bona fide aortic dissection, intimal tear or penetrating atherosclerotic ulcer. This process may arise from primary vasa vasorum haemorrhage within the aortic media or rupture of an atherosclerotic plaque [2]. IMH often displays a progression typical of aortic dissections and could be considered a precursor. IMH enfeebles the aortic wall along its entire longitudinal extension and may progress to either outward rupture of aorta or inward disruption of the intimal layer, which eventually leads to aortic dissection [3].

Penetrating aortic ulcer (PAU) is usually caused by ulceration of an aortic atherosclerotic lesion which penetrates the internal elastic media into the media [1] and radiologically manifests as a crater extending from the aortic lumen into the space surrounding the aortic lumen [2]. There are anecdotal reports of ascending aorta intramural hematoma secondary to descending aorta PAU, suggesting retrograde extension of intramural hematoma from descending aorta, at the site of PAU, to ascending aorta [4]. Equally there are anecdotal reports of intimal tears with flaps limited by transmural calcification of arch and descending aorta, mimicking PAU [5]. Histologically, aorta shows complete lack of elastic fibres at the level of the penetrating ulcer, with a dystrophic and thinned out aorta [6]. Although hypertension and atherosclerosis are present in majority of patients with PAU [1], PAU has been reported in patients with aortitis due to gonorrhoea [7, 8] and syphilis [9], following air travel [10], in immunosuppressed patients [11], in patients following CABG [12] and after inter-scapular blows to dislodge foreign bodies in oesophagus [13].

Taguchi et al. studied the effect of shear stress and atherosclerosis on intimal tear associated with aortic dissection and PAU in 30 patients over a 5 years period and found that high shear stress (greater curvature of aorta and anterior portion of aortic arch) and less severe atherosclerosis could induce the occurrence of an intimal tear and low shear stress and more severe atherosclerosis could proceed to PAU and IMH [14]. Elefteriades looked at PAU and IMH as pathological variants of classic aortic dissections and classified both PAU and IMH as non-flap lesions in contradistinction to aortic dissections which always involve a flap. They reported 36 out of 214 cases originally thought to be dissections and later found to be PAU or IMH and commented on their distinct clinical presentation. Compared to those with aortic dissections, Patients with PAU are distinctly older (74 v 65), invariably hypertensive (94%), do not produce branch vessel compromise or occlusion and ischaemic manifestations in visceral organs or extremities due to absence of longitudinal propagation, are most often associated with aortic arteriosclerosis and calcification, are present in larger aortas than classic dissection (6.2 cm vs 5.5 cm), are strongly associated with abdominal aortic aneurysms (42%), are largely diseases of descending aorta, are more prone to rupture and generally behave much more malignantly [1]. IMH too have a higher rupture rates, mainly due to the level of blood collection being more superficial and closer to adventitia than classic dissection [1].

In a review of 93 cases of PAU present in world literature at the time, Kodolitsch found 60% males, 85% with systemic hypertension, 31% with diabetes, 61% with associated coronary artery disease, 51% with thoracic or abdominal aortic aneurysm, 31% with chronic renal insufficiency, 17% with peripheral vascular disease and 12% with a history of cerebrovascular accidents [15]. 73% of PAUs were associated with medial hematoma and 16% with calcified intimal flap less than 10 cm. Sensitivities for demonstrating PAU were 83,65, 86 and 61% for angiography, CT, MRI and TOE respectively. 76% patients presented with chest or back pain, 8% with neurological signs like hoarseness, syncope or coma, 4% with embolic pulse differentials, 7% with aortic regurgitation, 42, 27 and 10% respectively with mediastinal hematoma, pleural and pericardial effusion. PAU of ascending aorta or arch (type A) led to dissection or rupture in 57%, compared to 12% in descending aorta. 57% of medically managed patients of type A PAU patients died within 30 days of hospital admission compared to only 14% of type B patients. On the basis of these findings, Kodolitsch et al. recommended type A PAU patients should undergo surgery while type B patients with PAU without signs of instability could be managed non- surgically [15].

Natural history of PAU of ascending aorta or arch is similar to type A dissection with the distinction that rupture [16–19] and pseudoaneurysm formation [20] seem to be much more common in PAU than in dissection. A contained rupture of PAU with extrinsic compression of pulmonary artery with right heart failure has been reported in a survivor [18] as have been occasional surgical salvages of ruptures of PAUs [17, 19]. In a retrospective review of 198 patients out of which 15 (7.6%) had PAU (2 in ascending aorta and 11 in descending aorta), Coady and Elefteriades found the risk of rupture to be highest in PAU (40%) compared with type A dissection (7%) or type B dissection (3.6%) and held the prognosis to be worse than dissection in aorta [16].

There is general agreement that PAUs of ascending aorta and arch behave in away similar to [15] or worse than type A dissection of aorta [16] and should have surgery as soon as possible [16–19, 21]. PAUs of descending aorta, although more prone to rupture, can be managed more conservatively, with TEVAR [22–26] or less frequently with expectant medical treatment [27] and more rarely with surgery with imminent or actual rupture [28]. Conversely, transapical TEVAR has been described for PAUs of ascending aorta, one with hybrid transangiographic aortic valve implantation (TAVI) for critical aortic stenosis [29] and the other on two successive occasions, second time for endovascular leak, followed eventually by interposition graft replacement of ascending aorta for persistent leak [30].

Coady and Elefteriades rereviewed 214 cases initially diagnosed as aortic dissection and found 36 cases (12%) to be either PAUs or IMHs. Compared with aortic dissections, patients having PAUs are older, more often hypertensive, more often having atherosclerosis of aorta or of visceral, cranial or limb arteries, and more often associated with larger aortas. Because PAUs are focal lesions which do not propagate vertically, they do not produce cerebral, visceral or limb ischemia due to branch vessel compromise. They found 90% of all PAUs to occur in descending aortas and found them to behave in a more malignant manner than descending aortic dissections [1].

Kodolitsch et al., in 1998, reported 93 cases of PAU in world literature and found that PAU of ascending aorta led to dissection and rupture in 57% and that of descending aorta in 5–12%. 57% of medically managed PAUs of ascending aorta or arch died within a month whilst only 14% of PAUs of descending aorta died within a month [15].

Henn et al. reported a 69 year old man with PAU of descending aorta with retrograde extension of intramural hematoma into ascending aorta, treated successfully by endovascular repair of the descending PAU and medical management of ascending aortic intramural hematoma [31].

Hetilap et al. found 18 patients with PAU out of 10,212 who underwent cardiac surgery (2 in ascending aorta and thoracoabdominal aorta each and 8 and 6 in aortic arch and descending aorta respectively). 4 patients had open surgery, 7 patients endografting and further 7 had hybrid operations with one hospital death and 2 late endoleaks.

Bernardes et al. reported better results in ascending aortic PAUs in contrast to ascending aortic dissections and reported their early experience with off-the-shelf endograft using a zone 0 landing site to treat ascending aorta and arch in 4 patients [23].

Fumikiyo et al. in a retrospective analysis of 65 patients with IMH with (34 patients) or without PAU (31 patients) found that IMH with PAU was mainly a descending aortic disease with only 9% present in ascending aorta as against 26% when unassociated with PAU. IMH with PAU was less stable with 48% progressing to more unstable disease as evidenced by sustained or recurrent pain, increasing pleural effusion and increasing maximum diameter of aorta and depth of PAU, as against 8% in those patients with IMU unassociated with PAU [32].

Cho et al. advocated a greater consideration for medical treatment of PAU of descending aorta or arch based on a 25 year review of 105 patients, out of which 76 patients had medical treatment and 29 patients surgery. Hospital mortality was 4 and 21% in medical and surgical groups respectively [27].

Only anecdotal reports exist of survivors following rupture of PAUs of thoracic aorta [18, 19, 24, 28, 33].

Yano reported a survivor of PAU rupture with aortic regurgitation and tamponade managed medically [33] and Okiweli described an octogenarian with contained rupture of PAU, with pulmonary trunk compression and transient pulmonary hypertension without obvious right heart failure, managed medically [18]. Lee reported ruptured PAU of ascending aorta treated with two parallel stent grafts [24]. Kovacevic described a dacron interposition graft replacement of an impending rupture of PAU of mid descending aorta with a 15 cm subadventitial haematoma [28]. Singhal reported successful management of a true rupture of PAU of ascending aorta with IMH and haemopericardium with surgery [19].

Our patient initially presented with four PAUs at distal ascending aorta and junction with the undersurface of arch, out of which the largest one measuring 2.5 × 1.5 cm had ruptured into a pseudo aneurysm which in turn ruptured into a large false aneurysmal sac which further ruptured into left pleural and pericardial cavity with about a litre of fresh blood in left pleural and pericardial cavities. This sequence of staggered rupture prevented sudden exsanguination. We performed an interposition graft replacement of ascending aorta and hemiarch employing unihemispherical antegrade selective cerebral perfusion (ASCP) at 17 C using sole innominate cannulation for establishing both cardiopulmonary bypass and providing ASCP, a technique we described earlier for bi-hemispherical ASCP in root and arch replacement in bovine arch variant anatomy in 2009 [34] and uni-hemispherical ASCP in acute type A dissection repair in patients with normal anatomy [35].

Patient was discharged having made good postoperative recovery but represented 6 months later in extremis with rupture of distal arch into a 14 cm false aneurysm compressing pulmonary trunk, left pulmonary artery, left recurrent laryngeal nerve and trachea causing extreme cachexia, hoarseness and acute respiratory embarrassment aggravated in certain postures. He underwent arch replacement with a 28 mm Vascutek dacron graft and 12 and 8 mm interposition grafts to IA and LCCA. The proximal anastomosis was made to the old graft replacing the ascending aorta and hemiarch and the distal anastomosis to the descending aorta. Cardiopulmonary bypass was established by composite right axillary arterial return supplemented on a Y connector by right femoral arterial cannulation and the venous drainage was established through femoral vein. Left brachiocephalic vein was divided for exposure, patient cooled to 17 C, and uni-hemispherical ASCP provided through the right axillary artery during the repair. He made satisfactory hemodynamic recovery from the second operation although respiratory wean due to his general frailty and resolving pneumonia was slower requiring percutaneous tracheostomy.

5 weeks later he developed yet another rupture downstream of proximal descending thoracic aorta with hypotension and respiratory distress and underwent TEVAR from which he made satisfactory recovery although he eventually succumbed to the complications of metastatic renal malignancy.

We have not come across any report in world literature of rupture of isolated PAU of ascending aorta who survived surgery except that described by Singhal which coexisted with IMH [19]. An aortic rupture of PAU is followed by exsanguination, unlike the occasional slow rupture of a dissection and these patients form subjects of postmortem findings. Our patient’s uniquely unusual staggered rupture first into a smaller pseudoaneurysm followed by rupture into a large false aneurysmal sac with subsequent rupture into both left pleural and pericardial cavities slowed the rate of exsanguination preventing sudden death.

Equally, survival with surgical correction following rupture of aortic arch secondary to atherosclerotic disease or PAU is unreported not to speak of such a course of events after previous ascending aorta and hemiarch replacement for ruptured PAU. Patient did not exsanguinate again because of slow rupture into a 14 cm aneurysm which slowed onset of hypovolaemic shock.

Further rupture of descending thoracic aorta again due to atherosclerosis or PAU was managed with TEVAR. There is a report of successful management of rupture of descending thoracic aorta due to PAU by Kovacevic although associated with sub-adventitial haematoma rather than frank rupture [28]. But generally descending thoracic aortic ruptures exsanguinate less comprehensively than ascending or arch ruptures due to the restraining effect of endothoracic fascia and therefore provide more time and opportunity for endovascular or even surgical redressal.

Could this sequence of events have been pre-empted? At the first operation, instead of replacing the ascending aorta and hemiarch only, one could have replaced the ascending aorta and total arch and performed a frozen trunk repair of the descending thoracic aorta. That would almost certainly have prevented further ruptures of arch and descending thoracic aorta over the next 8 months. However, the operative mortality and morbidity of doing an extensive and partially prophylactic procedure of additional total arch replacement and frozen elephant trunk repair in a patient who presented in extremis and in cardiogenic shock would have been prohibitively high. This would also involve presumption of hindsight. There was no way one could have predicted the highly unusual and almost unique course of events involving such an accelerated course of sequential ruptures of arch and descending thoracic aorta over a period as short as 8 months after the initial operation. The arch looked normal at the first operation once the ascending aorta and the under-surface of arch were excised.

The author and operating surgeon clearly made the decision of excising only that portion of aorta which was diseased and not the arch and descending aorta downstream prophylactically bearing the litany of Sir Robert Hutchison from the dictum of Sophocles in mind cautioning we should desist from the inability to let well alone, from too much zeal for what is new and contempt for what is old, from putting knowledge before wisdom, science before art, cleverness before common sense and above all from treating patients as cases and from making the cure of disease more grievous than its endurance. To which one could add the not so unknown hubris of doing something just because one can and not because it appears to be the need of the hour. The fact that patient would still have succumbed to the complications of metastatic renal malignancy is a different point altogether although the thought that somebody who braved three death-defying catastrophic complications of penetrating ulcers of aorta and the inevitable ordeals of the resultant salvage surgery then succumbed to completely unrelated metastatic renal malignancy was dismaying.

## Data Availability

Not applicable.

## Abbreviations

TEVAR: Thoracic endovascular aortic repair
AAS: Acute aortic syndrome
AD: Aortic Dissection
PAU: Penetrating aortic ulcer
CAR: Contained aortic rupture
AAA: Abdominal aortic aneurysm
IA: Innominate artery
LCCA: Left common carotid artery
LSA: Left subclavian artery
CT: Computed Tomography
MR: Magnetic Resonance
TOE: Transoesophageal echocardiography
TAVI: Transangiographic aortic valve implants
ASCP: Antegrade selective cerebral perfusion
